# Optimization of atmospheric leaching parameters for cadmium and zinc recovery from low-grade waste by response surface methodology (RSM)

**DOI:** 10.1038/s41598-024-52088-2

**Published:** 2024-01-17

**Authors:** Somayeh Kolbadinejad, Ahad Ghaemi

**Affiliations:** School of Chemical, Petroleum and Gas Engineering, Iran University of Science and Technology, Tehran, Iran

**Keywords:** Environmental sciences, Engineering

## Abstract

This study is focused on the optimization of effective parameters on Cadmium and Zinc recovery by atmospheric acid leaching of low-grade waste by response surface methodology (RSM) and using the Central Composite Design (CCD) method. The effects of parameters including time (0.5–2.5 h), temperature (40–80 °C), solid/liquid (S/L) (0.05–0.09 g/cc), particle size (174–44 mic), oxygen injection (0–1%) and pH (0.5–4.5) were statistically investigated at 5 surfaces. The sample of low-grade waste used in this study was mainly zinc factory waste. Two quadratic models for the correlation of independent parameters for the maximum recovery were proposed. The properties of waste were evaluated by X-ray diffraction (XRD) and X-ray fluorescence (XRF). Atomic absorption spectroscopy was used to determine the amount of Cadmium and Zinc in the leaching solution. The correlation coefficient (R^2^) for the predicted and experimental data of Cadmium and Zinc are 0.9837 and 0.9368, respectively. Time, S/L and size were the most effective parameters for the recovery efficiency of cadmium and zinc. 75.05% of Cadmium and 86.13% of Zinc were recovered in optimal conditions of leaching: S/L 0.08, pH 2.5, size 88 µm, 70 °C and 2.5 h. with air injection.

## Introduction

**Figure 1:**
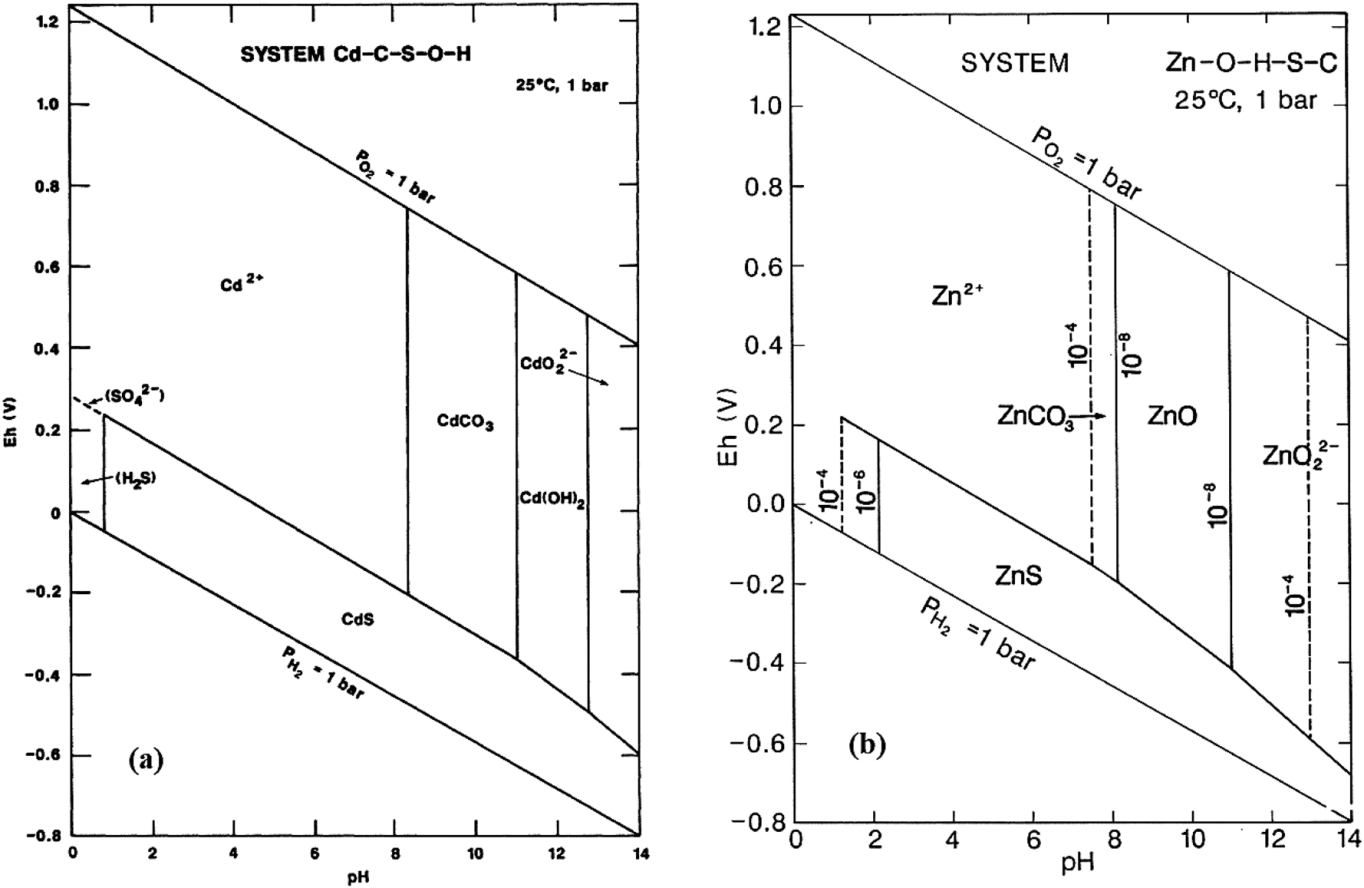
(**a**) Eh–pH diagram of the Cadmium–C–S–O–H system and (**b**) Eh–pH diagram of the Zinc–O–H–S–C system at 25°C and 1 bar.

Cadmium is a heavy and toxic metal in all kinds of slag, dust and liquid waste^1^ that enters the environment in the process of melting and extracting metals such as zinc^2^. Cadmium in its natural state and at the rate of 0.53 mg/kg in surface soil is not harmful to human health^3^, but due to the progress of industries, contamination of heavy metals in water and soil sources is more than allowed^4^ that its effects aren’t negligible^5^. Despite the zinc is needed for the human body, its large amount through oral, skin and inadvertent inhalation can affect the respiratory and gastrointestinal tracts as well as the brain with various side effects^6^. The increase in the high concentration of cadmium and zinc in water and soil sources is due to the combination of minerals with air and the release of metals^7,8^. The Eh–pH diagram of the Cadmium–C–S–O–H system and the Zinc–O–H–S–C system under various pH are shown in Fig. 1. As it can be seen that Cadmium^2+^ and Zinc^2+^ are present in an acidic environment, thermodynamically, it is possible to dissolve Cadmium and Zinc in an acidic environment^9^.

Acid leaching of heavy metals cadmium, zinc^10,11^, copper, lead and chromium is influenced by pH and mineral composition of slags^12^. Extractants such as: H_2_O, CH_3_COOH, HCl, EDTA, MgCl_2_ and NaCOOH are most used in separating metals from soil and waste^13^. The parameters of temperature, acid concentration, and time, solid / liquid (S/L) and stirring speed were investigated in acid leaching of zinc unit residues.

Selective and collective acid leaching is done to recover cadmium, zinc, nickel, lead and copper by H_2_SO_4_ 0.82 M^14^, 97% of cadmium was obtained from 8% (v/v) of H_2_SO_4_ 8% (v/v) leaching^15^, optimum acid leaching conditions were found for recovery of cadmium from zinc flue dust^16^, the recovery of hazardous materials by the leaching proposed in this research can be used industrially^17^, high leaching rate of cadmium, zinc and copper was achieved by oxidative acid leaching^18^, the leaching rate of cadmium reaches 99.63% by 110 g/L of H_2_SO_4_ 110 g/L^19^, the entire amount of cadmium, iron and cobalt and 95% of nickel were recovered during the acid leaching process^20^, in this article, first roasting and then hydrochloric acid leaching were used to separate zinc and other metals^21^, iminodiacetate aqueous solution was used for zinc recovery^22^.

H_2_SO_4_ concentration, along with other factors, is very important for cadmium and zinc leaching and has been investigated in various researches such as: 2 M H_2_SO_4_ with ultrasonic cavitation and mechanical effect^23^, 1.8 M H_2_SO_4_ under ultrasonic/ozone condition^24^, by using response surface methodology (RSM)–central composite design (CCD) modeling, temperature with agitation speed and liquid/solid ratio were the most effective factors on the recovery efficiency of zinc^25^, based on RSM, 94.3% of zinc was achieved under leaching optimal conditions^26^. In some researches, the S/L is the most effective parameter^14,27^, while in other studies, it has the opposite effect^15^. H_2_SO_4_ leaching using ultrasonic power of 300 W increases the efficiency recovery of zinc and cadmium^23,24^. Selective leaching of zinc from the cold filter cake was done in two steps with 8 M sodium hydroxide and finally 98% of zinc was separated, but nickel and cadmium remained in the solid residue. Separation of nickel and cadmium from the residue of the previous steps was carried out during acid leaching with 1 M H_2_SO_4_^28^. Alkali leaching of low-grade waste was investigated due to the lower cost for equipment. 85.52% of zinc was recovered with NaOH of 4 M^29^. Comparing the effect of nitric, hydrochloric, citric and H_2_SO_4_ on zinc leaching from low-grade waste, shows that the most dissolution of zinc occurs in nitric acid, and acid concentration and temperature are the most important parameters affecting the leaching process^30^. The implementation of laboratory results on a pilot scale can help to industrialize the optimal conditions obtained for the recovery of precious metals^31^. Response surface methodology is based on the design of experiments, includes mathematical and statistical tools, and optimizes the effective parameters of process^32,33^. Based on this, the recovery model of Cadmium and Zinc from the acid leaching process is determined by RSM^34^.

In this research, the effect of six parameters: time, temperature, S/L, particle size, oxygen injection and pH has been investigated on the simultaneous leaching of CADMIUM and Zinc from low-grade waste. Based on previous studies, the stirrer speed had almost no effect and was considered constant at 400 rpm with the aim of creating the required turbulence. Effective parameters were optimized by using Central Composite Design (CCD) with RSM. According to the use of H_2_SO_4_ at pH 2.5 instead of hydrochloric acid and nitric acid, the use of leaching versus pyrometallurgy at high temperatures and high efficiency for the simultaneous recovery of Cadmium and Zinc; the optimal conditions obtained from RSM significantly reduce energy consumption and Environmental pollution contributes. In addition, three scenarios checked in order to: 1. Minimum energy consumption, 2. Leaching without air injection and 3. Increasing the pH with reducing corrosion and preparing a suitable solution for the separation phase with industrial resin in the next step.

## Materials and method

### Materials

**Figure 2:**
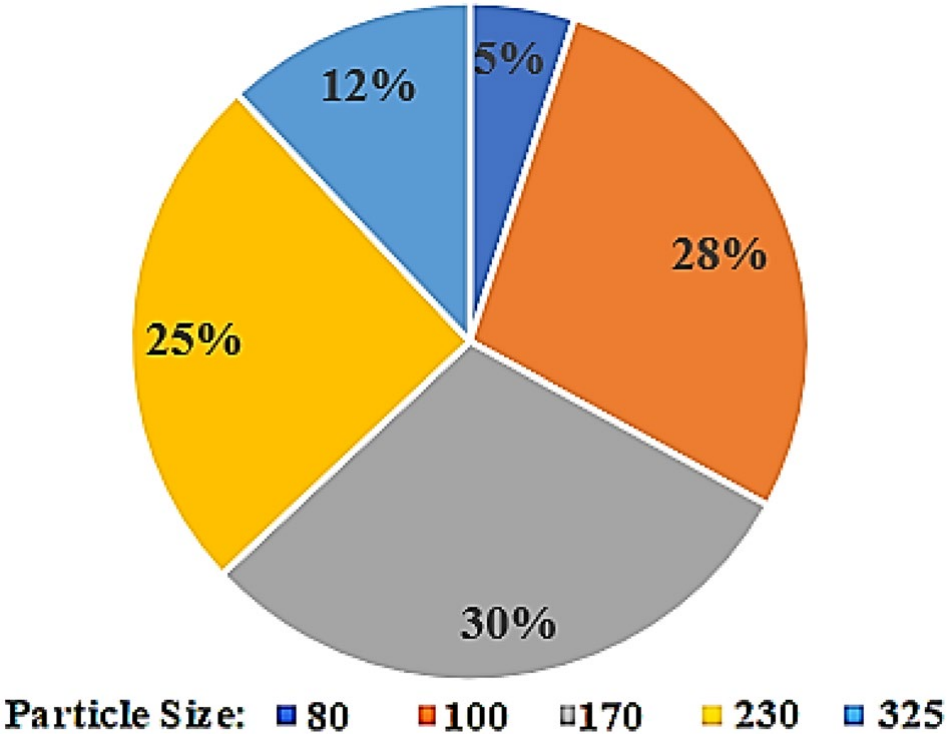
Particle size distribution of the sample used in the experiments.

The wastes of the zinc industry in Iran contain heavy and valuable metals such as cadmium and zinc. Releasing these wastes in nature is not allowed due to the high level of pollution and on the other hand; these wastes are secondary sources of supply for precious metals. In some units for metals recovery from waste, the recovery process is not done completely and the metals remaining in the waste are still higher than the permissible limit for releasing these wastes in nature, but these wastes are low-grade. Therefore, the simultaneous recovery of cadmium and zinc from low-grade wastes has been investigated in this research. About one kilogram of low-grade waste was first dehumidified in an oven at 200 °C for 12 h., then milled and granulated with sieves in sizes 80, 100, 170, 230 and 325 microns. Particles with different granulations were used for leaching experiments and particle size distribution is shown in Fig. 2.

Dehumidified and milled samples were analysed by X-ray diffraction (XRD) for the primary characterization of material properties like crystal structure, crystallite size, and X-ray fluorescence (XRF) for qualitative and quantitative analysis of material composition according to ASTM-E1621 standard. Laboratory H_2_SO_4_ and deionized water have been used.

### Methods

Performing leaching tests in optimal conditions requires checking the effect of effective parameters. According to the studies, the effect of time, temperature, solid–liquid ratio, particle size, injected oxygen and pH in the leaching of cadmium and zinc from low-grade waste was investigated, and 5 levels were considered for each parameter. 5 levels of each parameter are given in Table 1. According to the parameters and levels, the RSM suggested 52 tests. A report of tests conditions is provided in the supplementary in Table A.1.

**Table 1:** Independent parameters with their levels.

Independent parameters	Levels				
	−2	−1	0	1	2
Time (hr.)	0.5	1	1.5	2.0	2.5
Temperature (°C)	40	50	60	70	80
S/L (gr/cc)	7.5	9.0	10.5	12.0	13.5
Size (mic)	177	149	88	63	44
O_2_ Injection	0.0	0.25	0.5	0.75	1.0
	(Airless)	(Airless)	(With Air)	(With O3)	(With O3)
pH	0.5	1.5	2.5	3.5	4.5

The Central Composite Design (CCD) method is a widely used form of the response surface method (RSM) to optimize the leaching of valuable metals from industrial wastes^35,36^. This method evaluates the relative importance of effective factors and analyzes the mutual influence of these factors. Finally, this method optimizes the number of tests^25,26,34,37^. Equation (1) shows the quadratic polynomial, which is effective for predicting the optimal effects of the parameters.

1 $$y={\beta }_{0}+\sum_{i=1}{\beta }_{i}{X}_{i}+\sum_{i=1}{\beta }_{ij}{X}_{i}^{2}+\sum_{i=1}\sum_{j=i+1}{\beta }_{ij}{X}_{i}{X}_{j}+\varepsilon $$

where Y is the predicted response, β_0_ is the offset term, X_i_ and X_j_ are the independent variables, β_ii_ and β_ij_ are the interaction coefficients, respectively. ε is an unpredicted parameter that is determined experimentally. The cadmium or zinc extraction is calculating by Eq. (2):

2 $$\mathrm{Cadmium\, or\, Zinc\, Extraction }\left(\mathrm{\%}\right)= \frac{{X}_{2}- {X}_{1}}{{X}_{1}}\times 100$$

where X_2_ and X_1_ are the amount of cadmium or zinc after and before leaching (ppm), respectively.

The statistical criteriaof correlation coefficient (R^2^) was used to evaluate the accuracy and performance of the model, and the accuracy between the predicted values and the actual values^38^. R^2 ^was calculated as follows:

3 $${R}^{2}=\sum_{i=1}^{n}{({X}_{predicted}-{X}_{actual})}^{2}/{({X}_{predicted}-{X}_{mean})}^{2}$$

where, X_actual_ and X_predicted_ are the experimental and the predicted values by RSM, respectively. X_mean_ is mean value of data and n is number of data points.

#### Experiments

A three-necked glass reactor (150 cc) was used for leaching of dried filter cake with H_2_SO_4_. A thermometer, a line of air and condensation flow were fitted to three of the openings. The reactor was filled with a solution of H_2_SO_4_ and deionized water with a specific pH and then it was heated on the hot plate stirrer to reach the desired temperature. During the leaching experiments, the temperature was controlled to ± 5 °C. Stirring using a magnetic stirrer at a speed of 400 rpm was constant in all experiments. After the solution was reached to the specified temperature, the solid with the specific size and amount was added to the solution. For every experiment based on RSM, if needed, the flow of air has been injected with a certain flow rate. After the end of the test time, the separation of two solid and liquid phases was done with a Buchner funnel. The amount of cadmium and zinc in the leaching solution was measured with an atomic absorption spectroscopy (AAS/ model: WFX-220B).

**Figure 3:**
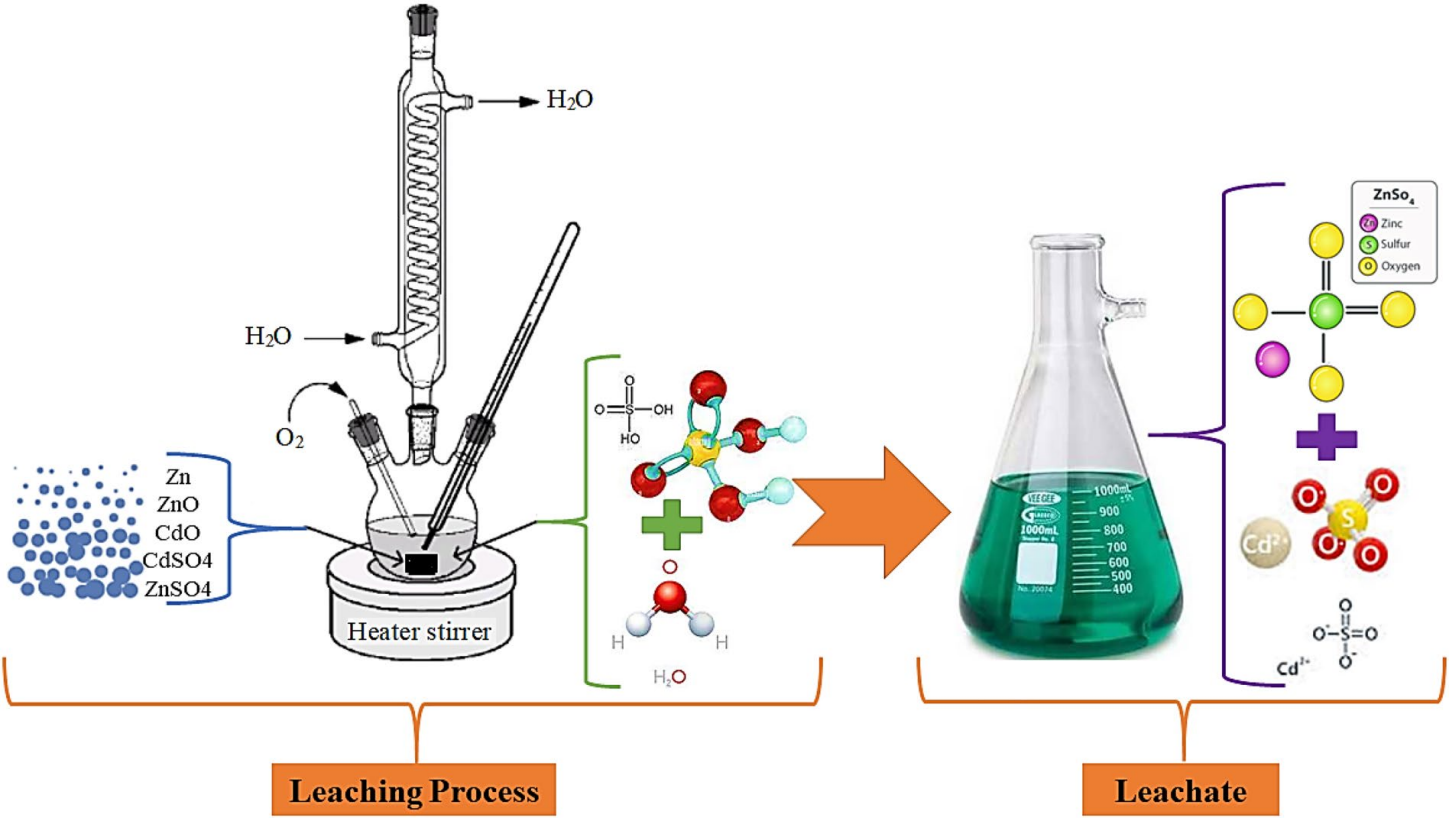
The schematic view of the experimental setup used in the current study.

The stirrer speed is not an effective parameter and can be kept constant to the extent of proper mixing of two phases. The complete leaching process is shown schematically in Fig. 3.

## Results and discussion

### Material characterization

**Figure 4:**
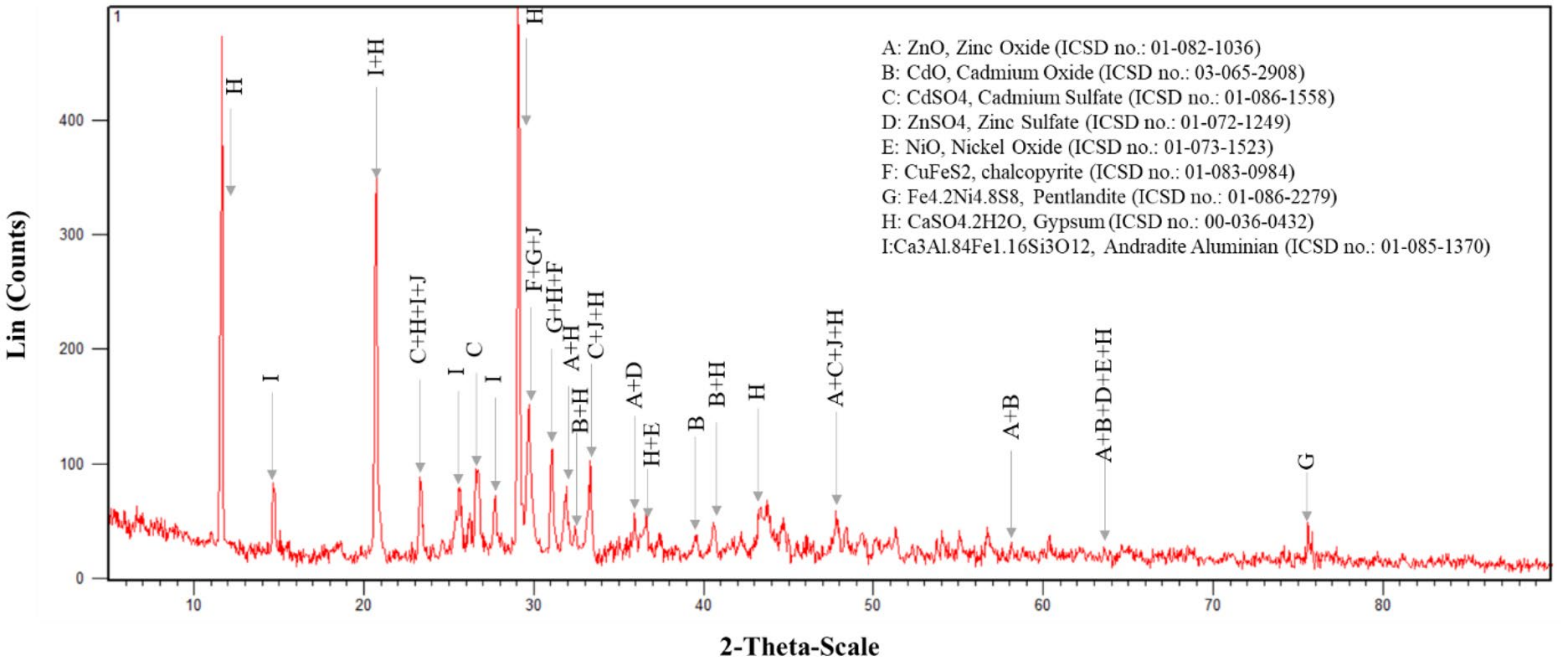
XRD analysis of the low-grade waste used in the study.

Determining the optimal conditions to increase the leaching efficiency requires the qualitative determination of the constituent phases of the materials and compounds in the primary waste, so XRD model D8 advance manufactured by Bruker Germany was used with a range of 2θ and a minimum scan step of 0.0001. The results show that Zn, ZnO, CdO, CdSO_4_, ZnSO_4_, CuFeS_2_, Ni_4_.5Fe_4_.5S_8_, CaSO_4_.2H_2_O, PbSO_4_ and Ca_3_Al.84Fe1.16Si_3_O_12_ were the most mineralogical phases in the low-grade waste. The XRD analysis of the low-grade waste used in the present research is shown in Fig. 4.

The elemental analysis of primary waste has been done by the XRF model PW1480 manufactured by PHILIPS equipped by X-ray lamp with a power of 3 kW. As can be seen in Table 2, the amounts of cadmium and zinc are about 2.59 and 1.74 wt.%, respectively, so it will be very difficult to recover them.

**Table 2:** The results of elemental analysis by X-ray fluorescence (XRF).

Element	SO_3_	CO_2_	CaO	SiO_2_	Al_2_O_3_	Cadmium	Na_2_O	Zinc	Fe_2_O_3_
wt%	36.101	24.400	12.676	7.6411	7.4960	2.5930	1.9571	1.7372	1.5362
Element	Pb	Ni	Mgo	Cu	Sr	Ge	Mn	Ti	Cr
wt%	1.4580	1.0445	0.73376	0.48484	0.1413	< <	< <	< <	< <

### Theory of leaching reactions

Attention to conducting experiments at the lowest possible temperature to reduce energy consumption and using simple equipment despite the use of H_2_SO_4_ has been considered in this research. As a result of the penetration of H_2_SO_4_ into the waste particles, the main reactions have taken place and the compounds of cadmium and zinc are dissolved according to Eqs. (4)–(7).

4 $$ Cd + H_{2} SO_{4} = CdSO_{4} + H_{2} \;\;\;\;\;\;\Delta G_{298}^{o} = - 31.76 kcal $$

5 $$ CdO + H_{2} SO_{4} = CdSO_{4} + H_{2} O\;\;\;\;\;\;\;\Delta G_{298}^{o} = - 33.631 kcal $$

6 $$ Zn + H_{2} SO_{4} = ZnSO_{4} + H_{2} \;\;\;\;\;\;\;\Delta G_{298}^{o} = - 43.64 kcal $$

7 $$ ZnO + H_{2} SO_{4} = ZnSO_{4} + H_{2} O\;\;\;\;\;\;\;\;\Delta G_{298}^{o} = - 23.747 kcal $$

Gibbs free energy is a thermodynamic quantity that shows the spontaneous rate of a reaction, but a reaction is thermodynamically possible when the changes in Gibbs free energy are negative. Therefore, the tendency to perform the reactions of Eqs. (4)–(7) is as follows:

8 $${R}_{Zn}>{R}_{Cd}$$

R represents the leaching efficiency. Based on the laboratory results, the recovery efficiency of zinc is higher than that of cadmium, which is a proof of the results confirmation.

Lead compounds do not dissolve in leaching conditions and are separated as residues. The reactions related to lead are given in Eq. (9). Also, in order to dissolve pentlandite (Fe9Ni9S16), roasting at high temperature and leaching under pressure is required, as a result of which iron oxide and nickel sulfide are obtained. But in the conditions of atmospheric leaching and without roasting pre-operation, dissolution of pentlandite (Fe9Ni9S16) does not happen (Eq. (10)).

9 $$Pb+{H}_{2}S{O}_{4} \to PbS{O}_{4}+{H}_{2}$$

10 $$2{Fe}_{9}{Ni}_{9}{S}_{16}+29{O}_{2}\to 18FeO+6{Ni}_{3}{S}_{2}+20S{O}_{2}$$

### RSM results

#### Regression model development

**Figure 5:**
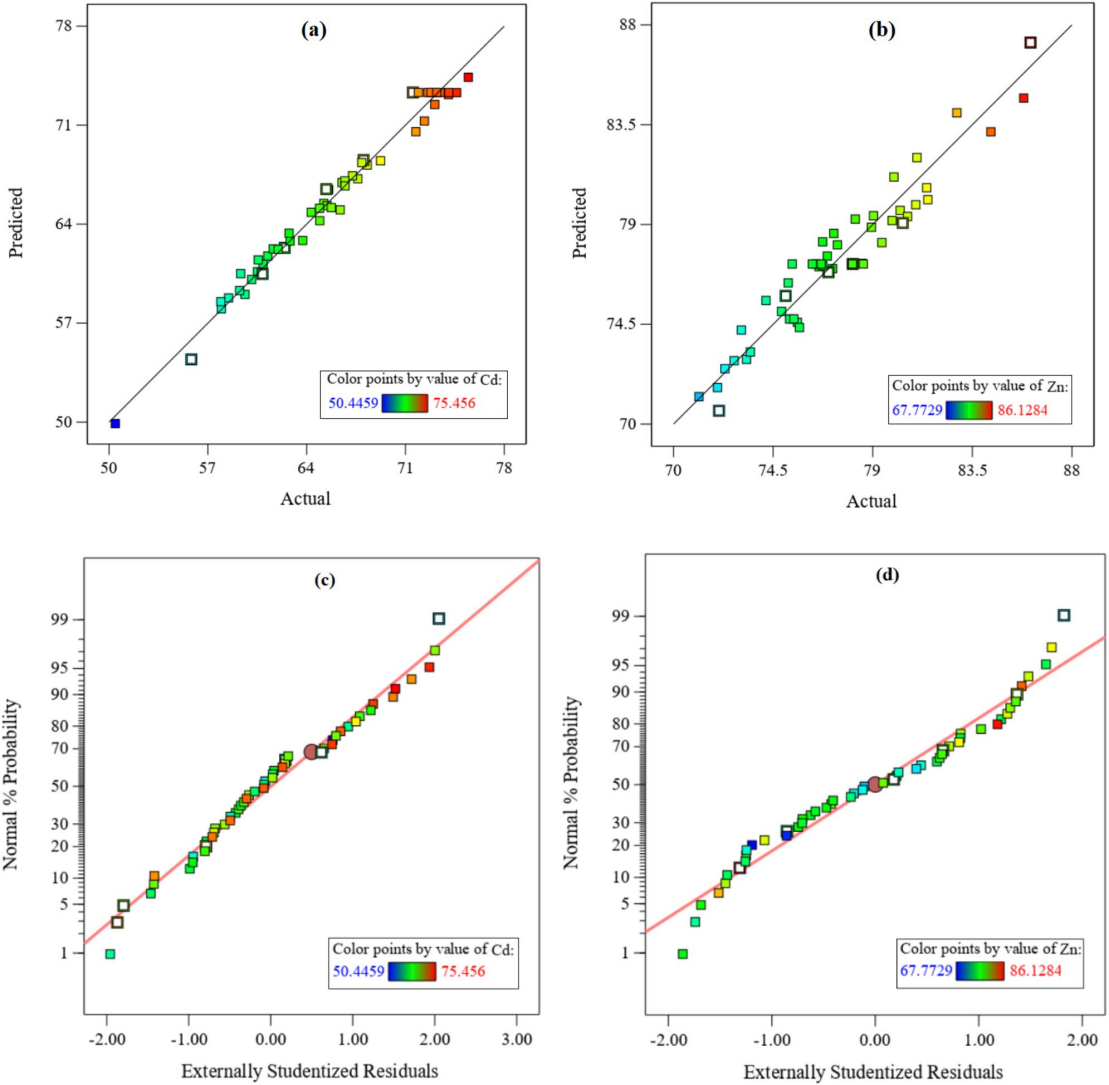
Predicted against actual values and Normal Plot of Residuals: (**a**) and (**c**) Cadmium recovery % and (**b**) and (**d**) Zinc recovery %.

According to the principles in RSM, models with *p*-value less than 0.05 and lack of fit value greater than 0.05 are acceptable. Based on the results of analysis of variance (ANOVA) for both results: (a) the *p*-value is less than 0.05 and (b) the lack of fit is greater than 0.05 and insignificant. Then, the analysis of variance of the Quadratic model is approved. The results are given in Appendix B. Table B.1 and B.2 for Cadmium and Zinc, respectively. The value of signal to noise ratio or adequate precision greater than 4 is desirable, which is 31.4004 and 17.7544 for cadmium and zinc, respectively. Actual and predicted values for Cadmium and Zinc recovery efficiency are given in Fig. 5a, b, respectively. According to Fig. 5, the values of R^2^ for cadmium and zinc are 0.9837 and 0.9368, respectively, and these values show that there is a good fit between the experimental data and the predicted values. All statistical values are presented in Table 3.

**Table 3:** Statistical parameters obtained from ANOVA for Cadmium and Zinc recovery on the quadratic regression model.

Metal recovery	Cadmium %	Zinc %
R^2^	0.9837	0.9368
Adequate precision	31.4004	17.7544
Standard deviation (SD)	1.06	1.41
Coefficient of variance (CV%)	1.62	1.82

The difference between the actual and predicted data is defined as the residual. If the results are on a straight line, it indicates that the error rate is normally distributed and the model is valid and approved. The residuals for normal are shown in Figs. 5c, d for Cadmium and Zinc, respectively.

#### Developed correlations

The coded equation is useful for identifying the relative impact of the factors by comparing the factor coefficients. The coefficient estimate represents the expected change in response per unit change in factor value when all remaining factors are held constant. By default, the high levels of the factors are coded as positive and the low levels are coded as negative. Quadratic models for cadmium and zinc recovery are based on Eqs. (11) and (12), respectively.

11 $$Cd\%=73.31+0.4667{\text{A}}+0.2113{\text{B}}+2.88{\text{C}}-1.14{\text{D}}-0.0394{\text{E}}-0.5147{\text{F}}-0.0359{\text{AB}}-0.0398{\text{AC}}+0.0626{\text{AD}}-0.0148{\text{AE}}-0.1647{\text{AF}}-0.0625{\text{BC}}-0.1727{\text{BD}}+0.0151{\text{BE}}+0.1424{\text{BF}}-0.0314{\text{CADMIUM}}-0.2813{\text{CE}}+0.0398{\text{CF}}-0.0069{\text{DE}}-0.2736{\text{DF}}-0.2720{\text{EF}}-0.2706{{\text{A}}}^{2}-0.1067{{\text{B}}}^{2}-1.17{{\text{C}}}^{2}-5.29{{\text{D}}}^{2}-2.05{{\text{E}}}^{2}-0.9487{{\text{F}}}^{2}.$$

12 $$  \begin{aligned} Zinc\% = & 77.22 + 0.6709A + 0.6435B + 2.32C - 0.6756D - 0.0978E - 0.7514F + 0.2495AB \\ & - 0.0272AC \, + 0.4575AD - 1.28AE + 1.15AF - 0.5080BC - 0.3575BD - 0.1148BE + 0.5584BF \\ & - 0.3875CD - 0.5715CE + 0.6389CF + 0.1368DE - 0.2124DF - 1.79EF + 1.53A + 0.5375B \\ & - 0.9194C + 0.1246D - 1.04E - 0.3690F \\ \end{aligned} $$

In these correlations, the coded terms of A, B, C, D, E and F are time, temperature, S/L, size, O_2_ injection and pH, respectively. The maximum and minimum value for recovery of cadmium and zinc from 52 designed experiments are 50.45–75.46 and 67.77–86.13, respectively.

As seen in Eq. (11), the constant coefficient of factor D (particle size) is larger than the others, but this factor has a negative sign, which means that this factor has the greatest effect on cadmium recovery. By increasing the size of solid particles, the opportunity for mass transfer and increasing the efficiency of cadmium leaching decreases. In Eq. (12), the constant coefficient of factor C (solid to liquid) is larger than the others, meaning that this factor has the greatest effect on zinc recovery. As the solid ratio increases, there is more opportunity for mass transfer and increased zinc leaching efficiency.

#### Analysis of RSM

**Figure 6:**
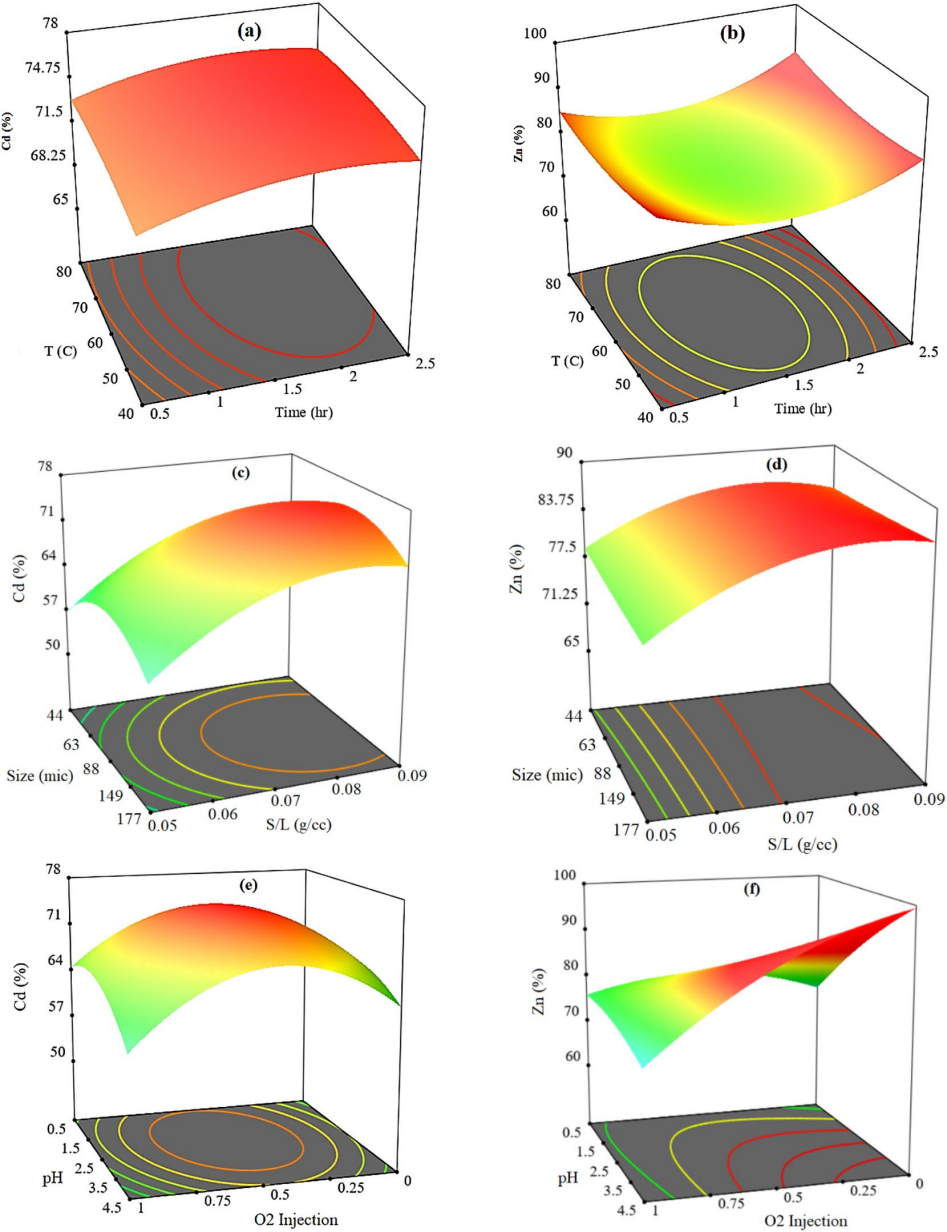
Response surfaces for (**a**) cadmium and (**b**) zinc recovery % versus Time and Temperature, (**c**) cadmium and (**d**) zinc recovery % versus S/L and Size, (**e**) cadmium and (**f**) zinc recovery % versus pH and O_2_ Injection.

Optimization criteria for the simultaneous recovery of cadmium and zinc from low-grade wastes have been investigated based on response surface methodology. Three-dimensional response surface plots show the interaction effect of parameters on the recovery efficiency of cadmium and zinc. Three-dimension response surface plots for Cadmium and Zinc recovery efficiency versus time and temperature are shown in Fig. 6a, b, respectively. The highest recovery efficiency is obtained at 70 °C and for 2.5 h. Increasing the temperature and time increases the surface area and the number of contacts between the two phases and then increases the mass transfer. Choosing a lower operating temperature is not recommended due to the significant reduction in recovery efficiency.

The effect of particle size and S/L ratio on the recovery efficiency of cadmium and zinc are shown in Fig. 6c, d, respectively. As the solid ratio increases for particles with size of 88 microns, the contact area increases and more mass transfer occurs. Particles with the smallest size have the least chance to mass transfer due to the reduced contact surface and more turbulence in the environment. Considering the significant changes in the recovery rate, the S/L ratio parameter is very effective in the recovery efficiency of Cadmium and Zinc. The changes of increasing S/L ratio on the recovery efficiency of cadmium are more than that of zinc.

The oxidizing agent is suitable for increasing the efficiency at pH 2.5 and with oxygen injection. But with the injection of oxygen (or air), the recovery efficiency increases due to the increase of turbulence in the experiment environment and the reduction of contact time between phases. Figure 6e, f show the recovery efficiency for cadmium and zinc as a function of oxygen injection and pH parameters, respectively.

Depending on the range of variables, the optimal values for each variable are in the Table 4.

**Table 4:** The optimal values of variables to recover cadmium and zinc.

Variables	Time (hr.)	Temperature (°C)	S/L (g/cc)	Size (mic)	O_2_ Injection	pH	Cadmium %	Zinc %
Amount	2.5	70	12	88	0.5	2.5	75.05	86.13

### Effect of effective parameters

**Figure 7:**
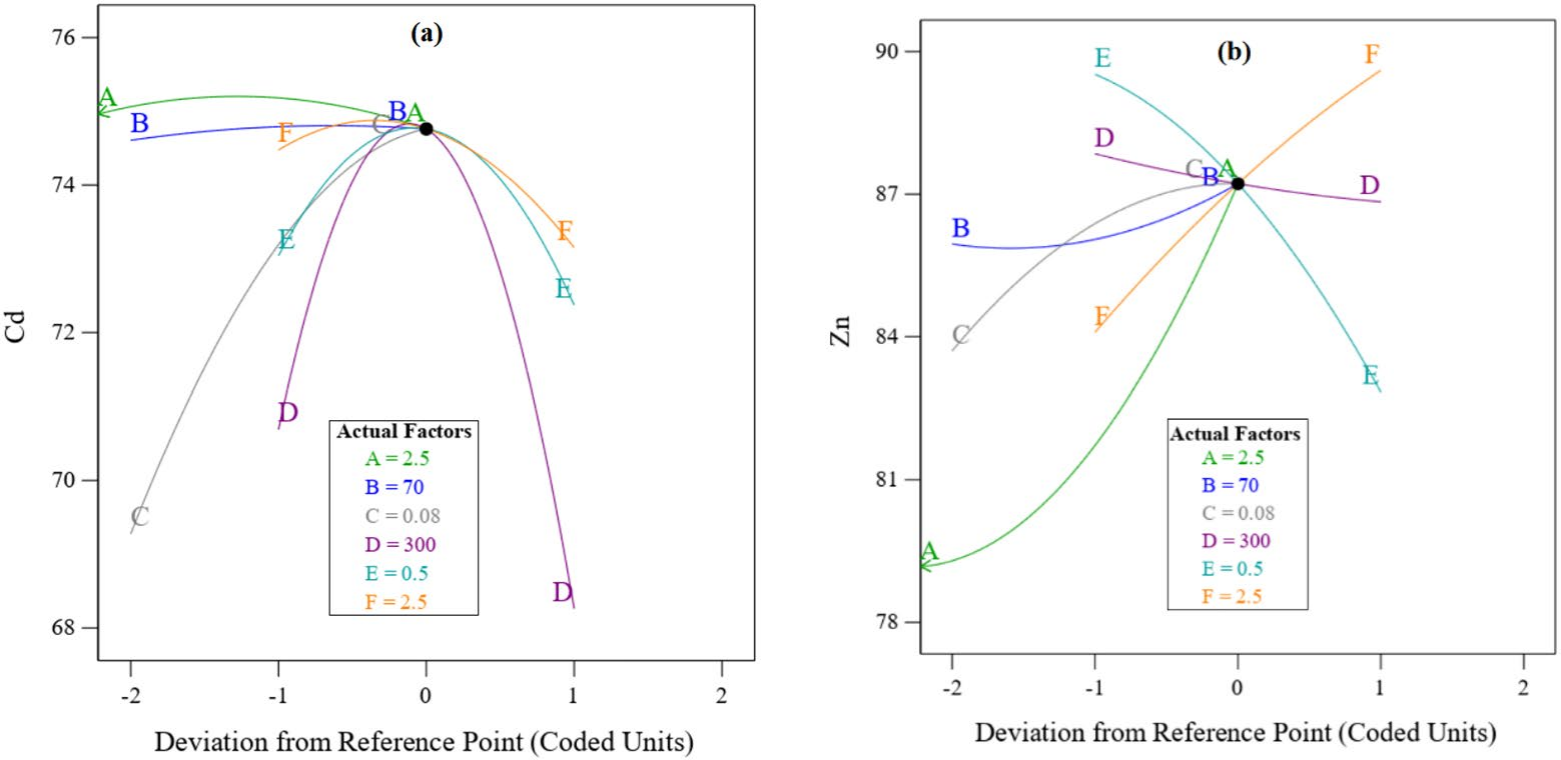
Effect of Effective Parameters on (**a**) Cadmium recovery % and (**b**) Zinc recovery %. Actual factors are: A: Time, B: Temperature, C: S/L, D: Size, E: O_2_ Injection and F: pH.

The effect of the parameters has been investigated in the conditions where the maximum recovery for cadmium and zinc is considered. Particle size, S/L, and oxygen injection are the most effective parameters on cadmium recovery efficiency. According to Fig. 7a, the contact surface increases by the particle size increasing to 88 µm but at the particle size larger than 88 µm, the movement of the particles decreases and the efficiency also decreases. But always with the increase of S/L due to the increase of the contact surface, the efficiency increases. The effect of effective parameters in the recovery of zinc show in Fig. 7b. Time, oxygen injection, pH and S/L are the most effective parameters on zinc recovery efficiency. By increasing the time, pH and S/L, there is enough oxidizing agent and opportunity to increase the contacts and mass transfer.

Also, for recovery efficiency of cadmium and zinc, oxygen injection increases the efficiency due to increasing the appropriate contacts, and then decreases the efficiency by increasing the turbulency and reducing the necessary contact time. [Rewrite the text].

## Optimization

Three scenarios have been considered for optimization with the aim of 1: minimum energy consumption, 2: leaching without air injection and 3. Increasing the pH.

### Minimum energy consumption

**Figure 8:**
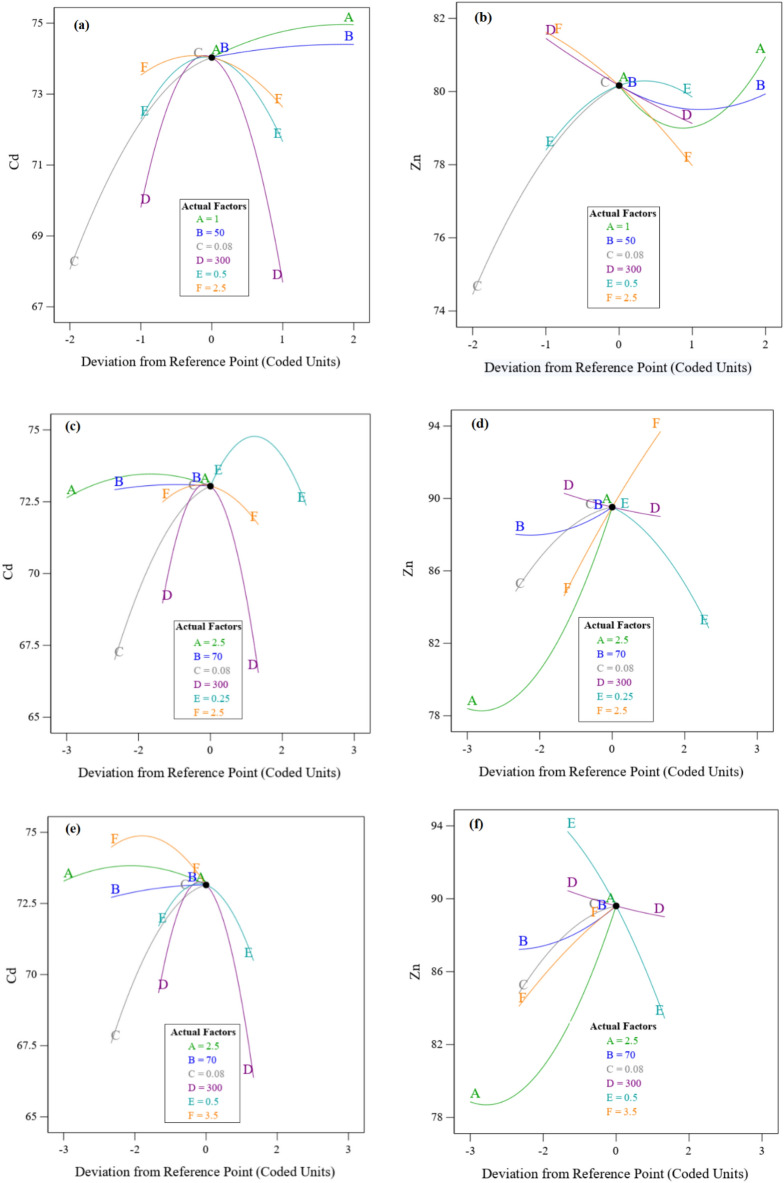
Optimization scenarios for Cadmium% and Zinc%. Actual factors are: A: Time, B: Temperature, C: S/L, D: Size, E: O_2_ Injection and F: pH.

In order to minimize energy consumption, it is necessary in optimal conditions to change the values of parameters affecting energy consumption, such as time and temperature. By examining different modes in temperature changes from 40 to 80 °C and time from 0.5 h. to 2.5 h., the result has been obtained that by changing the temperature from the optimal value of 70 to 50 °C and the time from the optimal value of 2.5 h. to 0.5 h., the recovery efficiency of Cadmium and Zinc has changed from 75.05% and 86.13% to 74.03% and 80.17%, respectively. Reducing the time and temperature will reduce the contact of two phases and ultimately mass transfer, and for this reason, the efficiency has decreased, but reducing the temperature by 20 °C along with 2 h. in the industrial unit will reduce the energy consumption. The effect of different parameters is shown in Fig. 8a, b for Cadmium and Zinc, respectively. With the increase of S/L, the recovery efficiency of Cadmium and Zinc increases due to the increase in the number of contacts, but firstly by increasing in particle size, pH and oxygen injection, the Cadmium recovery increases and then decreases, while the increase in particle size and pH for Zinc recovery has a decreasing trend because the movement of larger particles is reduced and mass transfer is also reduced. In these circumstances, particle size and S/L in Cadmium recovery and S/L and time in Zinc recovery are the most effective parameters.

### Leaching without air injection

This scenario is considered without oxygen (air) injection because the cost of air pump and fittings is reduced. The parameters of time, temperature, S/L and particle size were kept constant in optimal values that are given in Table 4. As shown in Fig. 8c, d, the Zinc and Cadmium recovery efficiency has significantly changed from 86.13% to 89.51% and 75.05% to 73.05, respectively. Increasing time, temperature and S/L have an increasing effect on the efficiency of Zinc and Cadmium due to the increase in the number of contacts and mass transfer, but changes in particle size first have an increasing effect and then a decreasing effect. Air injection also has a reducing effect on Zinc efficiency.

### Increasing the pH

As the pH increases, the acidic state of the leaching solution decreases, which has advantages such as: reduced corrosion, less acid consumption, and more suitable conditions for the separation of metals by industrial resins in the next step. All parameters except pH are similar to optimal conditions that are given in the Table 4, but the value of pH is increased from 2.5 to 3.5 and versus Fig. 8e, f, the Cadmium efficiency has decreased from 75.05% to 73.15 but the Zinc recovery efficiency has increased significantly from 86.13 to 89.61%, respectively. Increasing time, temperature, S/L, and pH have an increasing effect on the efficiency of Zinc and Cadmium due to the increase in the number of contacts and mass transfer, but changes in particle size and air injection have an increasing and then decreasing effect.

## Conclusion

Reduction of resources, need of industries and prevention of environmental pollution are the main reasons for recovering cadmium and zinc from low-grade waste by hydrometallurgy and pyrometallurgy at high temperature and pressure. Single-stage atmospheric leaching has been used to recover valuable, heavy and toxic metals cadmium and zinc from low-grade waste. The maximum recovery efficiency in optimal leaching conditions with the use of RSM in conjunction with CCD has been discussed. Regression analysis and optimization of parameters were used to predict the response in the experimental regions. Statistical analysis of the experiments result performed under different conditions of the parameters. Two models were obtained for the effective parameters in the leaching of cadmium and zinc. Based on models, particle size, S/L, and oxygen injection are the most effective parameters on cadmium recovery efficiency. Time, oxygen injection, pH and S/L are the most effective parameters on zinc recovery efficiency. By increasing the particle size, time, pH and S/L, there is enough oxidizing agent and opportunity to increase the contacts and mass transfer. Also, Oxygen injection increases the efficiency due to increasing the appropriate contacts. 75.05% of cadmium and 86.13% of zinc were recovered during the leaching process in 2.5 h., temperature of 70 °C, 12 g of solid particles of 88 µm in 150 cc solution with pH 2.5 and with oxygen injection. Considering the efficiency and suitable operating conditions, it is suggested to implement the process on a semi-industrial and industrial scale.

### Supplementary Information

Supplementary Information 1.

Supplementary Information 2.

## Data Availability

The datasets used and analysed during the current study available from the corresponding author on reasonable request.
