# Supplementary material for: Optimization of atmospheric leaching parameters for cadmium and zinc recovery from low-grade waste by response surface methodology (RSM)
Source: Sci Rep. 2024 Jan 17;14:1490. doi: 10.1038/s41598-024-52088-2 (PMC10794212; doi:10.1038/s41598-024-52088-2)
Supplement: Supplementary file 2 — Supplementary Information 2. [file 41598_2024_52088_MOESM2_ESM.docx]

**Supplementary**

**Table A.1 –** Independent factors and responses through experimental design by RSM modelling.

| **Run** | **Factors** | | | | | | | **Response 1** | **Response 2** |
| --- | --- | --- | --- | --- | --- | --- | --- | --- | --- |
|  | **Time (hr.)** | **Temp. (C)** | **S/L (g/cc)** | **Size (mic)** | **E** | | **pH** | **Cd%** | **Zn%** |
| 1 | 1.5 | 60 | 10.5 | 177 | 0.50 | Air | 2.5 | 55.83 | 80.36 |
| 2 | 2.0 | 70 | 12.0 | 63 | 0.25 | Airless | 1.5 | 65.56 | 76.74 |
| 3 | 1.0 | 50 | 9.0 | 149 | 0.75 | O_2_ | 3.5 | 60.87 | 72.06 |
| 4 | 2.0 | 70 | 12.0 | 149 | 0.25 | Airless | 3.5 | 68.03 | 86.13 |
| 5 | 2.0 | 70 | 9.0 | 149 | 0.75 | O_2_ | 3.5 | 61.98 | 75.24 |
| 6 | 1.5 | 60 | 10.5 | 88 | 0.50 | Air | 4.5 | 69.23 | 73.06 |
| 7 | 2.0 | 70 | 9.0 | 149 | 0.25 | Airless | 1.5 | 62.44 | 75.07 |
| 8 | 1.5 | 60 | 10.5 | 88 | 0.50 | Air | 2.5 | 73.45 | 78.08 |
| 9 | 1.0 | 50 | 12.0 | 63 | 0.25 | Airless | 1.5 | 65.22 | 76.57 |
| 10 | 2.0 | 50 | 12.0 | 149 | 0.75 | O_2_ | 3.5 | 65.39 | 77.00 |
| 11 | 2.5 | 60 | 10.5 | 88 | 0.50 | Air | 2.5 | 74.05 | 85.82 |
| 12 | 1.5 | 60 | 10.5 | 88 | 0.50 | Air | 2.5 | 71.52 | 78.09 |
| 13 | 1.5 | 60 | 10.5 | 88 | 0.50 | Air | 2.5 | 71.91 | 76.44 |
| 14 | 1.0 | 70 | 12.0 | 63 | 0.25 | Airless | 3.5 | 64.32 | 77.19 |
| 15 | 2.0 | 50 | 9.0 | 63 | 0.75 | O_2_ | 3.5 | 58.46 | 71.15 |
| 16 | 1.0 | 70 | 9.0 | 149 | 0.75 | O_2_ | 1.5 | 61.63 | 79.96 |
| 17 | 1.0 | 50 | 9.0 | 149 | 0.25 | Airless | 1.5 | 59.33 | 73.30 |
| 18 | 2.0 | 70 | 9.0 | 63 | 0.75 | O_2_ | 1.5 | 60.57 | 76.95 |
| 19 | 2.0 | 70 | 12.0 | 63 | 0.75 | O_2_ | 3.5 | 64.93 | 76.74 |
| 20 | 1.5 | 60 | 10.5 | 88 | 1.00 | O_2_ | 2.5 | 66.38 | 72.74 |
| 21 | 1.5 | 60 | 10.5 | 88 | 0.50 | Air | 2.5 | 74.06 | 76.27 |
| 22 | 1.0 | 50 | 12.0 | 149 | 0.25 | Airless | 3.5 | 67.62 | 80.23 |
| 23 | 1.0 | 50 | 12.0 | 63 | 0.75 | O_2_ | 3.5 | 62.76 | 75.43 |
| 24 | 2.0 | 50 | 9.0 | 149 | 0.75 | O_2_ | 1.5 | 62.83 | 74.88 |
| 25 | 1.5 | 60 | 13.5 | 88 | 0.50 | Air | 2.5 | 75.46 | 79.4148 |
| 26 | 1.5 | 40 | 10.5 | 88 | 0.50 | Air | 2.5 | 73.08 | 77.40 |
| 27 | 2.0 | 70 | 9.0 | 63 | 0.25 | Airless | 3.5 | 59.24 | 80.57 |
| 28 | 1.0 | 50 | 9.0 | 63 | 0.75 | O_2_ | 1.5 | 60.10 | 80.94 |
| 29 | 0.5 | 60 | 10.5 | 88 | 0.50 | Air | 2.5 | 72.34 | 80.99 |
| 30 | 1.5 | 60 | 10.5 | 88 | 0.50 | Air | 2.5 | 73.01 | 78.57 |
| 31 | 2.0 | 70 | 12.0 | 149 | 0.75 | O_2_ | 1.5 | 67.91 | 79.87 |
| 32 | 2.0 | 50 | 9.0 | 63 | 0.25 | Airless | 1.5 | 60.52 | 75.69 |
| 33 | 2.0 | 50 | 9.0 | 149 | 0.25 | Airless | 3.5 | 60.91 | 74.18 |
| 34 | 1.0 | 50 | 9.0 | 63 | 0.25 | Airless | 3.5 | 57.96 | 68.53 |
| 35 | 2.0 | 50 | 12.0 | 63 | 0.25 | Airless | 3.5 | 65.45 | 84.34 |
| 36 | 1.0 | 70 | 9.0 | 149 | 0.25 | Airless | 3.5 | 61.25 | 75.59 |
| 37 | 1.5 | 80 | 10.5 | 88 | 0.50 | Air | 2.5 | 74.63 | 81.44 |
| 38 | 1.0 | 70 | 12.0 | 149 | 0.75 | O_2_ | 3.5 | 66.71 | 77.24 |
| 39 | 1.0 | 70 | 9.0 | 63 | 0.75 | O_2_ | 3.5 | 57.92 | 71.99 |
| 40 | 2.0 | 50 | 12.0 | 149 | 0.25 | Airless | 1.5 | 68.29 | 79.03 |
| 41 | 1.0 | 70 | 12.0 | 149 | 0.25 | Airless | 1.5 | 67.25 | 78.94 |
| 42 | 1.5 | 60 | 10.5 | 88 | 0.50 | Air | 2.5 | 72.58 | 78.05 |
| 43 | 1.5 | 60 | 7.5 | 88 | 0.50 | Air | 2.5 | 63.72 | 67.77 |
| 44 | 1.5 | 60 | 10.5 | 88 | 0.50 | Air | 0.5 | 71.74 | 78.52 |
| 45 | 1.0 | 70 | 12.0 | 63 | 0.75 | O_2_ | 1.5 | 64.91 | 81.49 |
| 46 | 1.0 | 50 | 12.0 | 149 | 0.75 | O_2_ | 1.5 | 66.50 | 82.80 |
| 47 | 1 | 70 | 9 | 63 | 0.25 | Airless | 1.5 | 59.63 | 72.32 |
| 48 | 1.5 | 60 | 10.5 | 44 | 0.5 | Air | 2.5 | 50.46 | 75.18 |
| 49 | 2 | 50 | 12 | 63 | 0.75 | O_2_ | 1.5 | 66.72 | 78.21 |
| 50 | 1.5 | 60 | 10.5 | 88 | 0.5 | Air | 2.5 | 73.22 | 76.67 |
| 51 | 1.5 | 60 | 10.5 | 88 | 0 | Airless | 2.5 | 65.75 | 73.47 |
| 52 | 1.5 | 60 | 10.5 | 88 | 0.5 | Air | 2.5 | 72.80 | 75.36 |

**Appendix B**

**Table B.1 –** Detail of ANOVA information for all parameters and their interaction effects.

| **Source** | **Cd %** | | | | | **Zn %** | | | | |
| --- | --- | --- | --- | --- | --- | --- | --- | --- | --- | --- |
|  | **S.S.** | **df** | **M.S.** | **FV** | **PV** | **S.S.** | **df** | **M.S.** | **FV** | **PV** |
| **Model** | 1639.68 | 27 | 60.73 | 53.75 | < 0.0001 | 702.15 | 27 | 26.01 | 13.17 | < 0.0001 |
| A-Time | 8.71 | 1 | 8.71 | 7.71 | 0.0105 | 18.00 | 1 | 18.00 | 9.12 | 0.0059 |
| B-T | 1.79 | 1 | 1.79 | 1.58 | 0.2206 | 16.57 | 1 | 16.57 | 8.39 | 0.0079 |
| C-S/L | 332.87 | 1 | 332.87 | 294.63 | < 0.0001 | 215.52 | 1 | 215.52 | 109.17 | < 0.0001 |
| D-Size | 51.60 | 1 | 51.60 | 45.68 | < 0.0001 | 18.26 | 1 | 18.26 | 9.25 | 0.0056 |
| E-E | 0.0622 | 1 | 0.0622 | 0.0550 | 0.8165 | 0.3828 | 1 | 0.3828 | 0.1939 | 0.6636 |
| F-pH | 10.60 | 1 | 10.60 | 9.38 | 0.0053 | 22.58 | 1 | 22.58 | 11.44 | 0.0025 |
| AB | 0.0413 | 1 | 0.0413 | 0.0366 | 0.8500 | 1.99 | 1 | 1.99 | 1.01 | 0.3252 |
| AC | 0.0508 | 1 | 0.0508 | 0.0450 | 0.8339 | 0.0238 | 1 | 0.0238 | 0.0120 | 0.9136 |
| AD | 0.1254 | 1 | 0.1254 | 0.1110 | 0.7419 | 6.70 | 1 | 6.70 | 3.39 | 0.0779 |
| AE | 0.0070 | 1 | 0.0070 | 0.0062 | 0.9380 | 52.33 | 1 | 52.33 | 26.51 | < 0.0001 |
| AF | 0.8678 | 1 | 0.8678 | 0.7681 | 0.3895 | 42.68 | 1 | 42.68 | 21.62 | 0.0001 |
| BC | 0.1249 | 1 | 0.1249 | 0.1105 | 0.7424 | 8.26 | 1 | 8.26 | 4.18 | 0.0519 |
| BD | 0.9540 | 1 | 0.9540 | 0.8444 | 0.3673 | 4.09 | 1 | 4.09 | 2.07 | 0.1629 |
| BE | 0.0073 | 1 | 0.0073 | 0.0064 | 0.9367 | 0.4215 | 1 | 0.4215 | 0.2135 | 0.6482 |
| BF | 0.6489 | 1 | 0.6489 | 0.5743 | 0.4559 | 9.98 | 1 | 9.98 | 5.05 | 0.0340 |
| CD | 0.0315 | 1 | 0.0315 | 0.0278 | 0.8689 | 4.80 | 1 | 4.80 | 2.43 | 0.1319 |
| CE | 2.53 | 1 | 2.53 | 2.24 | 0.1474 | 10.45 | 1 | 10.45 | 5.30 | 0.0304 |
| CF | 0.0506 | 1 | 0.0506 | 0.0448 | 0.8342 | 13.06 | 1 | 13.06 | 6.62 | 0.0167 |
| DE | 0.0015 | 1 | 0.0015 | 0.0014 | 0.9709 | 0.5986 | 1 | 0.5986 | 0.3032 | 0.5870 |
| DF | 2.39 | 1 | 2.39 | 2.12 | 0.1584 | 1.44 | 1 | 1.44 | 0.7314 | 0.4009 |
| EF | 2.37 | 1 | 2.37 | 2.10 | 0.1606 | 102.81 | 1 | 102.81 | 52.08 | < 0.0001 |
| A² | 2.42 | 1 | 2.42 | 2.14 | 0.1563 | 77.77 | 1 | 77.77 | 39.39 | < 0.0001 |
| B² | 0.3758 | 1 | 0.3758 | 0.3327 | 0.5695 | 9.54 | 1 | 9.54 | 4.83 | 0.0378 |
| C² | 45.47 | 1 | 45.47 | 40.25 | < 0.0001 | 27.92 | 1 | 27.92 | 14.14 | 0.0010 |
| D² | 922.92 | 1 | 922.92 | 816.90 | < 0.0001 | 0.5131 | 1 | 0.5131 | 0.2599 | 0.6148 |
| E² | 139.40 | 1 | 139.40 | 123.39 | < 0.0001 | 35.81 | 1 | 35.81 | 18.14 | 0.0003 |
| F² | 29.73 | 1 | 29.73 | 26.31 | < 0.0001 | 4.50 | 1 | 4.50 | 2.28 | 0.1443 |
| **Residual** | 27.11 | 24 | 1.13 |  |  | 47.38 | 24 | 1.97 |  |  |
| Lack of Fit | 22.41 | 17 | 1.32 | 1.96 | 0.1856 | 38.08 | 17 | 2.24 | 1.69 | 0.2470 |
| Pure Error | 4.71 | 7 | 0.6726 |  |  | 9.30 | 7 | 1.33 |  |  |
| **Cor Total** | 1666.79 | 51 |  |  |  | 749.53 | 51 |  |  |  |
| S.S.= Sum of Squares, M.S.= Mean Square, FV= F-value, PV= p-value | | | | | | | | | | |

**Appendix C**

**Table C.1 –** Statistical data about developed correlations based on ANOVA.

| \| **Factor** \| **Cd %** \| \| \| \| \| \| \| --- \| --- \| --- \| --- \| --- \| --- \| --- \| \| **Coefficient Estimate** \| **df** \| **Standard Error** \| **95% CI Low** \| **95%**  **CI**  **High** \| **VIF** \| \| Intercept \| 73.31 \| 1 \| 0.3515 \| 72.58 \| 74.03 \|  \| \| A-Time \| 0.4667 \| 1 \| 0.1681 \| 0.1199 \| 0.8136 \| 1.0000 \| \| B-T \| 0.2113 \| 1 \| 0.1681 \| -0.1355 \| 0.5582 \| 1.0000 \| \| C-S/L \| 2.88 \| 1 \| 0.1681 \| 2.54 \| 3.23 \| 1.0000 \| \| D-Size \| -1.14 \| 1 \| 0.1681 \| -1.48 \| -0.7890 \| 1.0000 \| \| E-E \| -0.0394 \| 1 \| 0.1681 \| -0.3863 \| 0.3074 \| 1.0000 \| \| F-pH \| -0.5147 \| 1 \| 0.1681 \| -0.8616 \| -0.1679 \| 1.0000 \| \| AB \| -0.0359 \| 1 \| 0.1879 \| -0.4237 \| 0.3519 \| 1.0000 \| \| AC \| -0.0398 \| 1 \| 0.1879 \| -0.4276 \| 0.3480 \| 1.0000 \| \| AD \| 0.0626 \| 1 \| 0.1879 \| -0.3252 \| 0.4504 \| 1.0000 \| \| AE \| -0.0148 \| 1 \| 0.1879 \| -0.4026 \| 0.3730 \| 1.0000 \| \| AF \| -0.1647 \| 1 \| 0.1879 \| -0.5525 \| 0.2231 \| 1.0000 \| \| BC \| -0.0625 \| 1 \| 0.1879 \| -0.4503 \| 0.3253 \| 1.0000 \| \| BD \| -0.1727 \| 1 \| 0.1879 \| -0.5605 \| 0.2151 \| 1.0000 \| \| BE \| 0.0151 \| 1 \| 0.1879 \| -0.3727 \| 0.4029 \| 1.0000 \| \| BF \| 0.1424 \| 1 \| 0.1879 \| -0.2454 \| 0.5302 \| 1.0000 \| \| CD \| -0.0314 \| 1 \| 0.1879 \| -0.4192 \| 0.3564 \| 1.0000 \| \| CE \| -0.2813 \| 1 \| 0.1879 \| -0.6691 \| 0.1065 \| 1.0000 \| \| CF \| 0.0398 \| 1 \| 0.1879 \| -0.3480 \| 0.4276 \| 1.0000 \| \| DE \| -0.0069 \| 1 \| 0.1879 \| -0.3947 \| 0.3809 \| 1.0000 \| \| DF \| -0.2736 \| 1 \| 0.1879 \| -0.6614 \| 0.1142 \| 1.0000 \| \| EF \| -0.2720 \| 1 \| 0.1879 \| -0.6598 \| 0.1158 \| 1.0000 \| \| A² \| -0.2706 \| 1 \| 0.1849 \| -0.6523 \| 0.1111 \| 1.01 \| \| B² \| -0.1067 \| 1 \| 0.1849 \| -0.4884 \| 0.2750 \| 1.01 \| \| C² \| -1.17 \| 1 \| 0.1849 \| -1.56 \| -0.7916 \| 1.01 \| \| D² \| -5.29 \| 1 \| 0.1849 \| -5.67 \| -4.90 \| 1.01 \| \| E² \| -2.05 \| 1 \| 0.1849 \| -2.44 \| -1.67 \| 1.01 \| \| F² \| -0.9487 \| 1 \| 0.1849 \| -1.33 \| -0.5670 \| 1.01 \| | \| **Factor** \| **Zn %** \| \| \| \| \| \| \| --- \| --- \| --- \| --- \| --- \| --- \| --- \| \| **Coefficient Estimate** \| **df** \| **Standard Error** \| **95% CI Low** \| **95%**  **CI**  **High** \| **VIF** \| \| Intercept \| 77.22 \| 1 \| 0.4647 \| 76.26 \| 78.18 \|  \| \| A-Time \| 0.6709 \| 1 \| 0.2222 \| 0.2124 \| 1.13 \| 1.0000 \| \| B-T \| 0.6435 \| 1 \| 0.2222 \| 0.1850 \| 1.10 \| 1.0000 \| \| C-S/L \| 2.32 \| 1 \| 0.2222 \| 1.86 \| 2.78 \| 1.0000 \| \| D-Size \| -0.6756 \| 1 \| 0.2222 \| -1.13 \| -0.2171 \| 1.0000 \| \| E-E \| -0.0978 \| 1 \| 0.2222 \| -0.5563 \| 0.3607 \| 1.0000 \| \| F-pH \| -0.7514 \| 1 \| 0.2222 \| -1.21 \| -0.2928 \| 1.0000 \| \| AB \| 0.2495 \| 1 \| 0.2484 \| -0.2632 \| 0.7621 \| 1.0000 \| \| AC \| -0.0272 \| 1 \| 0.2484 \| -0.5399 \| 0.4854 \| 1.0000 \| \| AD \| 0.4575 \| 1 \| 0.2484 \| -0.0552 \| 0.9701 \| 1.0000 \| \| AE \| -1.28 \| 1 \| 0.2484 \| -1.79 \| -0.7661 \| 1.0000 \| \| AF \| 1.15 \| 1 \| 0.2484 \| 0.6423 \| 1.67 \| 1.0000 \| \| BC \| -0.5080 \| 1 \| 0.2484 \| -1.02 \| 0.0046 \| 1.0000 \| \| BD \| -0.3575 \| 1 \| 0.2484 \| -0.8702 \| 0.1551 \| 1.0000 \| \| BE \| -0.1148 \| 1 \| 0.2484 \| -0.6274 \| 0.3979 \| 1.0000 \| \| BF \| 0.5584 \| 1 \| 0.2484 \| 0.0457 \| 1.07 \| 1.0000 \| \| CD \| -0.3875 \| 1 \| 0.2484 \| -0.9001 \| 0.1252 \| 1.0000 \| \| CE \| -0.5715 \| 1 \| 0.2484 \| -1.08 \| -0.0589 \| 1.0000 \| \| CF \| 0.6389 \| 1 \| 0.2484 \| 0.1262 \| 1.15 \| 1.0000 \| \| DE \| 0.1368 \| 1 \| 0.2484 \| -0.3759 \| 0.6494 \| 1.0000 \| \| DF \| -0.2124 \| 1 \| 0.2484 \| -0.7250 \| 0.3002 \| 1.0000 \| \| EF \| -1.79 \| 1 \| 0.2484 \| -2.31 \| -1.28 \| 1.0000 \| \| A² \| 1.53 \| 1 \| 0.2445 \| 1.03 \| 2.04 \| 1.01 \| \| B² \| 0.5375 \| 1 \| 0.2445 \| 0.0329 \| 1.04 \| 1.01 \| \| C² \| -0.9194 \| 1 \| 0.2445 \| -1.42 \| -0.4148 \| 1.01 \| \| D² \| 0.1246 \| 1 \| 0.2445 \| -0.3799 \| 0.6292 \| 1.01 \| \| E² \| -1.04 \| 1 \| 0.2445 \| -1.55 \| -0.5366 \| 1.01 \| \| F² \| -0.3690 \| 1 \| 0.2445 \| -0.8735 \| 0.1356 \| 1.01 \| |
| --- | --- | --- | --- | --- | --- | --- | --- | --- | --- | --- | --- | --- | --- | --- | --- | --- | --- | --- | --- | --- | --- | --- | --- | --- | --- | --- | --- | --- | --- | --- | --- | --- | --- | --- | --- | --- | --- | --- | --- | --- | --- | --- | --- | --- | --- | --- | --- | --- | --- | --- | --- | --- | --- | --- | --- | --- | --- | --- | --- | --- | --- | --- | --- | --- | --- | --- | --- | --- | --- | --- | --- | --- | --- | --- | --- | --- | --- | --- | --- | --- | --- | --- | --- | --- | --- | --- | --- | --- | --- | --- | --- | --- | --- | --- | --- | --- | --- | --- | --- | --- | --- | --- | --- | --- | --- | --- | --- | --- | --- | --- | --- | --- | --- | --- | --- | --- | --- | --- | --- | --- | --- | --- | --- | --- | --- | --- | --- | --- | --- | --- | --- | --- | --- | --- | --- | --- | --- | --- | --- | --- | --- | --- | --- | --- | --- | --- | --- | --- | --- | --- | --- | --- | --- | --- | --- | --- | --- | --- | --- | --- | --- | --- | --- | --- | --- | --- | --- | --- | --- | --- | --- | --- | --- | --- | --- | --- | --- | --- | --- | --- | --- | --- | --- | --- | --- | --- | --- | --- | --- | --- | --- | --- | --- | --- | --- | --- | --- | --- | --- | --- | --- | --- | --- | --- | --- | --- | --- | --- | --- | --- | --- | --- | --- | --- | --- | --- | --- | --- | --- | --- | --- | --- | --- | --- | --- | --- | --- | --- | --- | --- | --- | --- | --- | --- | --- | --- | --- | --- | --- | --- | --- | --- | --- | --- | --- | --- | --- | --- | --- | --- | --- | --- | --- | --- | --- | --- | --- | --- | --- | --- | --- | --- | --- | --- | --- | --- | --- | --- | --- | --- | --- | --- | --- | --- | --- | --- | --- | --- | --- | --- | --- | --- | --- | --- | --- | --- | --- | --- | --- | --- | --- | --- | --- | --- | --- | --- | --- | --- | --- | --- | --- | --- | --- | --- | --- | --- | --- | --- | --- | --- | --- | --- | --- | --- | --- | --- | --- | --- | --- | --- | --- | --- | --- | --- | --- | --- | --- | --- | --- | --- | --- | --- | --- | --- | --- | --- | --- | --- | --- | --- | --- | --- | --- | --- | --- | --- | --- | --- | --- | --- | --- | --- | --- | --- | --- | --- | --- | --- | --- | --- | --- | --- | --- | --- | --- | --- | --- | --- | --- | --- | --- | --- | --- | --- | --- | --- | --- | --- | --- | --- | --- | --- | --- | --- | --- | --- | --- | --- | --- | --- | --- | --- | --- | --- | --- | --- | --- | --- | --- | --- | --- | --- | --- | --- | --- | --- | --- | --- | --- | --- | --- | --- | --- | --- | --- | --- | --- | --- | --- |
